# A Deep Learning–Based Rotten Food Recognition App for Older Adults: Development and Usability Study

**DOI:** 10.2196/55342

**Published:** 2024-07-03

**Authors:** Minki Chun, Ha-Jin Yu, Hyunggu Jung

**Affiliations:** 1 Department of Computer Science and Engineering University of Seoul Seoul Republic of Korea; 2 Department of Artificial Intelligence University of Seoul Seoul Republic of Korea

**Keywords:** digital health, mobile health, mHealth, app, apps, application, applications, smartphone, smartphones, classification, digital sensor, deep learning, artificial intelligence, machine learning, food, foods, fruit, fruits, experience, experiences, attitude, attitudes, opinion, opinions, perception, perceptions, perspective, perspectives, acceptance, adoption, usability, gerontology, geriatric, geriatrics, older adult, older adults, elder, elderly, older person, older people, ageing, aging, aged, camera, image, imaging, photo, photos, photograph, photographs, recognition, picture, pictures, sensor, sensors, develop, development, design

## Abstract

**Background:**

Older adults are at greater risk of eating rotten fruits and of getting food poisoning because cognitive function declines as they age, making it difficult to distinguish rotten fruits. To address this problem, researchers have developed and evaluated various tools to detect rotten food items in various ways. Nevertheless, little is known about how to create an app to detect rotten food items to support older adults at a risk of health problems from eating rotten food items.

**Objective:**

This study aimed to (1) create a smartphone app that enables older adults to take a picture of food items with a camera and classifies the fruit as rotten or not rotten for older adults and (2) evaluate the usability of the app and the perceptions of older adults about the app.

**Methods:**

We developed a smartphone app that supports older adults in determining whether the 3 fruits selected for this study (apple, banana, and orange) were fresh enough to eat. We used several residual deep networks to check whether the fruit photos collected were of fresh fruit. We recruited healthy older adults aged over 65 years (n=15, 57.7%, males and n=11, 42.3%, females) as participants. We evaluated the usability of the app and the participants’ perceptions about the app through surveys and interviews. We analyzed the survey responses, including an after-scenario questionnaire, as evaluation indicators of the usability of the app and collected qualitative data from the interviewees for in-depth analysis of the survey responses.

**Results:**

The participants were satisfied with using an app to determine whether a fruit is fresh by taking a picture of the fruit but are reluctant to use the paid version of the app. The survey results revealed that the participants tended to use the app efficiently to take pictures of fruits and determine their freshness. The qualitative data analysis on app usability and participants’ perceptions about the app revealed that they found the app simple and easy to use, they had no difficulty taking pictures, and they found the app interface visually satisfactory.

**Conclusions:**

This study suggests the possibility of developing an app that supports older adults in identifying rotten food items effectively and efficiently. Future work to make the app distinguish the freshness of various food items other than the 3 fruits selected still remains.

## Introduction

### Background

Older adults over the age of 65 years need a system that supports distinguishing rotten food items because older adults are exposed to the danger of eating rotten food and suffer from health problems [1]. Compared to younger age groups, older adults have a lower ability to recognize whether food items are rotten [2], and as they get older, their cognitive function decreases [3], which causes a proportionate overall decrease in visual, olfactory, and gustatory functions [4].

Prior studies have found that decreased olfactory function increases the chances of eating rotten foods. According to a study by the University of Pennsylvania Medical School, olfactory disorders affect the quality of life, appetite, and weight [5]. Similarly, the results of the National Health and Nutrition Examination Survey in the United States found that people over the age of 70 years have difficulty recognizing dangerous odors, such as those of smoke and gas [6]. As such, older adults are in danger of being exposed to food poisoning if they ingest rotten food items due to the difficulty detecting whether the items have gone bad. Food poisoning is caused by the ingestion of toxic substances, as well as bacteria contained in rotten food items [7]. To address this danger, researchers have developed and evaluated tools to detect rotten food items [8,9] for unspecified users or specific users, such as people with olfactory impairment. Prior studies have also used chemical sensors and kits and, in some cases, cameras to detect rotten food items and measure freshness [10].

### Rotten Food Item–Detecting Tools Targeted at Unspecified Users

Prior studies have proposed and evaluated tools to measure food freshness for unspecified users [8-21]. Researchers have used chemical sensors or kits to detect the spoilage of specific food items such as pork and chicken [11-14] or unspecified food items [10,15,16], such as ham [11], fruits (eg, banana, grape) [12], and pork [13]. Specifically, to detect spoilage of ham produced in Spain, Choi et al [11] proposed a tool that determines whether ham is rotten using a sensor that responds to sulfur-containing compounds in the gas generated during ham decay. In addition, Caya et al [12] proposed a tool with an electronic nose to detect gas generated during the decay of bananas, carrots, and grapes by using k-nearest neighbors and principal component analysis of the collected data. Similarly, Tian et al [13] proposed a tool that detects spoilage of pork by determining the number of aerobic bacteria in pork through a sensor and principal component analysis. Meanwhile, Mikš‐Krajnik et al [14] proposed a tool detect 27 kinds of volatile organic compounds generated during the decay of chickens by selecting 3 types of indicators highly correlated with spoilage in order to detect spoilage of chickens. Researchers have also proposed tools with an electronic nose to detect decay using gas generated from meat, fruits, and vegetables [10,16]. Meanwhile, Janagama et al [15] used an organism detection kit called a dipstick to detect wine spoilage.

Researchers have also proposed various methods (eg, convolutional neural network [CNN], Fourier transform infrared spectroscopy [FTIR]) to detect the spoilage of specific food items using a camera [8,9,17-21]. Perez-Daniel et al [17] used a camera to detect the spoilage of unspecified food items. They proposed a tool that collects images of both normal and rotten food items through a neural network using RetinaNet and compared them to detect the food spoilage. Other researchers have proposed tools to detect the spoilage of certain food items, such as fruits [8,18,19], vegetables [9], beef [20], and rice [21]. For example, Karakaya et al [8] obtained a co-occurrence matrix from a grayscale histogram of an image to detect decay in apples, bananas, and oranges and extracted features from the obtained matrix using the bag-of-feature method. The extracted features were classified into normal food items and rotten food items using a CNN and a support vector machine (SVM). In addition, researchers have proposed tools to detect decay in citrus fruits [19] and other fruits [18] using visual features. Jagtap et al [9] proposed a tool to take pictures of potatoes moving through a conveyor belt and detect decay in them using a neural network and features extracted from the pictures. Meanwhile, Ellis et al [20] used FTIR and machine learning to detect food spoilage through chemical changes in beef. Additionally, Batugal et al [21] proposed a tool to detect decay in Philippine rice using both a chemical sensor and a camera; this tool uses machine learning to classify data collected into spoiled rice and normal rice [21].

### Rotten Food Item–Detecting Tools Targeted at Specific Users

Prior studies have proposed and evaluated tools to measure food freshness for specific users, such as people who manage the freshness of meats [22-26]. These tools detect food spoilage, even in unspecified food items [22], using chemical sensors or kits [23-25]. Specifically, researchers have proposed and evaluated tools to determine the spoilage of tomato dishes [24], fish and beef [24], and milk [25] using a gas sensor. Researchers who proposed and evaluated a tool to detect spoilage of tomato dishes in the Philippines using an electronic nose for people with an olfactory impairment equipped with a methane/CH_4_ quality (MQ) gas sensor and a temperature and humidity sensor to reduce the spoilage of tomato dishes [23]. Similarly, a tool for identifying the spoilage of fish and beef using an electronic nose, an artificial neural network, an SVM, and k-nearest neighbors was proposed for food freshness inspection by butchers [24]. Another study proposed a tool for identifying spoiled milk using an electronic nose and fuzzy c-means clustering to prevent older adults suffering from olfactory disorders and dementia from drinking spoiled milk [25]. Musa et al [22] proposed a film for packaging material developers that changes color when it encounters rotten food items by sensing the pH with corn starch-glycerol and anthocyanin.

A previous study proposed a tool for certain users to use a chemical sensor and a camera to determine food spoilage. Kodogiannis et al [26] proposed a tool that detects microorganisms in meat using FTIR and an advanced clustering-based neuro-fuzzy identification model, determining the degree of meat spoilage.

### Limitations of Prior Studies

Nevertheless, little is known about studies that have developed and evaluated apps to help determine the freshness of fruits for the basic diet of physically healthy older adults. Therefore, we developed and evaluated a smartphone app for healthy older adults that determines whether apples, bananas, and oranges are rotten. Apples, bananas, and oranges are among the most consumed traditional fruits in South Korea and the United States [27]. In addition, we could easily collect photos of rotten apples, bananas, and oranges, so we selected them as the target fruits of our app [28]. Textbox 1 shows the research questions of our study.

By answering our research questions, we will contribute to older adults and the community of researchers. First, we proposed a smartphone app that supports older adults in avoiding consuming rotten fruits. Second, we revealed their perceptions about the app and showed them how to evaluate the app, which uses an artificial intelligence (AI) model. Third, we contributed to the related research community by revealing which of the 7 pretrained backbone networks we used to classify rotten fruits showed the best performance. To answer the research questions, we reviewed the relevant literature and developed and evaluated the performance of the app. To evaluate the app, we used an after-scenario questionnaire [29] and conducted semistructured interviews [30] with older adults.

**Research questions.**

- Research question 1: What studies have previously evaluated and developed an app for healthy older adults to take pictures of food items with a smartphone camera to determine whether the items are rotten?
- Research question 2: Is it possible to make an app that classifies apples, bananas, and oranges with only their pictures taken with a smartphone camera?
  Research question 2.1: How can we gather pictures that are needed to create a function for classifying apples, bananas, and oranges?
  Research question 2.2: How can we create a function to determine whether apples, bananas, and oranges are rotten?
  Research question 2.3: How can we create an app that can use the aforementioned functions?
- Research question 3: Is there a significant performance difference between functions created by various methods of classifying apples, bananas, and oranges?
- Research question 4: Is the app that determines whether an apple, a banana, or an orange is rotten only by using pictures taken with a smartphone camera easy to use?
  Research question 4.1: Why are older adults satisfied with the process of using the app?
  Research question 4.2: Why are older adults dissatisfied with the process of using the app?
  Research question 4.3: Why are older adults satisfied with the time it takes to complete the task?
  Research question 4.4: Why are older adults dissatisfied with the time taken to complete the task?
  Research question 4.5: What makes older adults satisfied using the app?
  Research question 4.6: What makes older adults dissatisfied with using the app?
  Research question 4.7: What do older adults want from using the app?
- Research question 5: What are the perceptions about the app that determines whether an apple, a banana, or an orange is rotten?
  Research question 5.1: Who are the potential users of the app?
  Research question 5.2: Why did older adults suggest them as potential users?
  Research question 5.3: Why can older adults trust the app?
  Research question 5.4: Why can older adults not trust the app?
  Research question 5.5: What features would older adults like to add to the app?
  Research question 5.6: Why do older adults use the app even if it is a paid app?

## Methods

### Study Design

This study aimed to develop and evaluate a smartphone app that enables older adults to take pictures of food items with a smartphone camera and to determine whether the items are rotten. We first reviewed prior studies that have developed and evaluated similar spoilage detection tools. After reviewing the relevant literature, we trained a model that classifies the freshness of 3 fruits (ie, apples, bananas, and oranges) and evaluated the model’s performance. Next, we developed a smartphone app that uses the trained model to take images of fruits and classify them into images of fresh and rotten fruits. We recruited 23 older adult participants to evaluate the usability of the app and to determine their perceptions about the app.

### Literature Review

**Figure 1:**
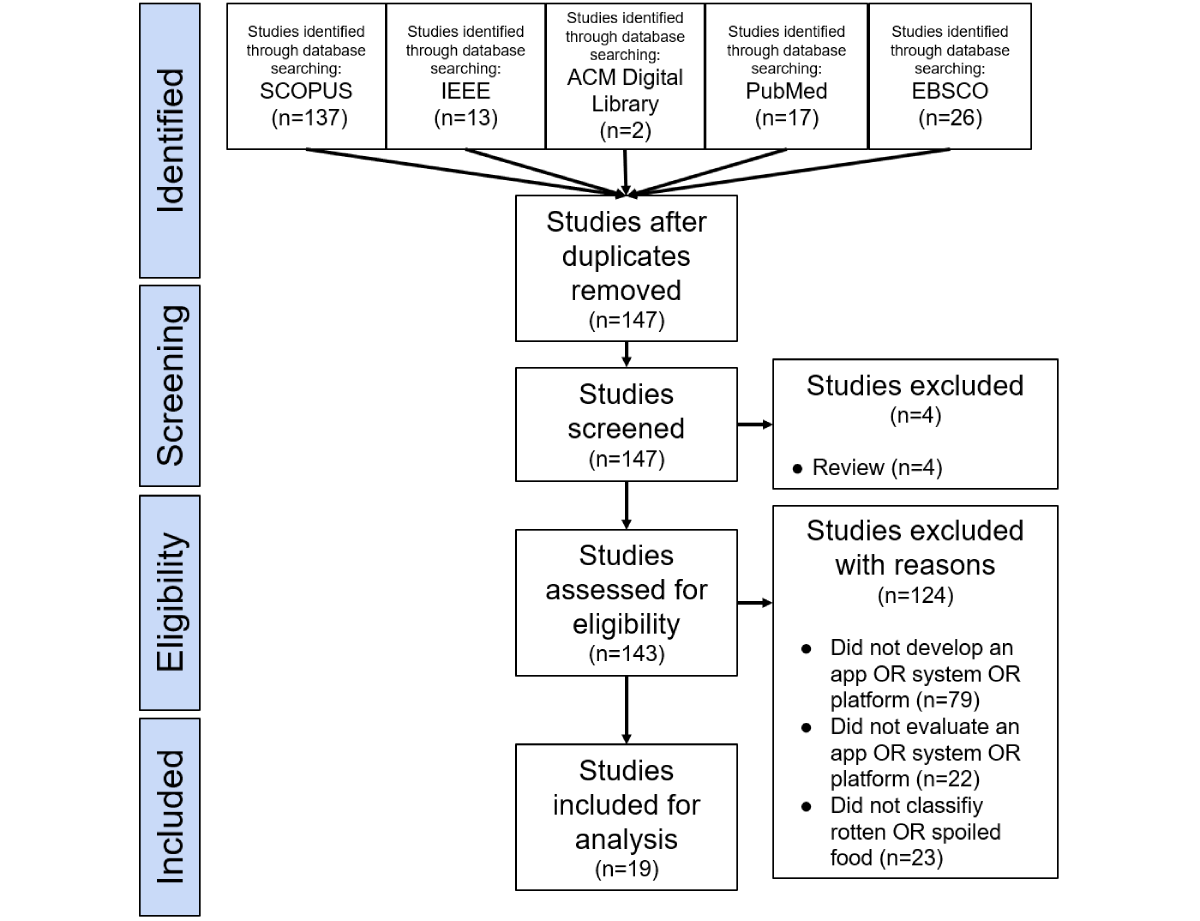
PRISMA flow diagram for reviewing prior studies. After deduplicating the papers in each database, we screened review papers and found 19 that met the eligibility criteria. PRISMA: Preferred Reporting Items for Systematic Reviews and Meta-Analyses.

The purpose of the literature review was to discover the limitations of prior studies that have developed and evaluated tools that classify food items into rotten and fresh items. To collect prior studies that have reported any developed apps determining food freshness by taking pictures of food items, we used a combination of keywords (eg, “rotten,” “food,” “collect,” and “app”) in Scopus, IEEE, the ACM Digital Library, PubMed, and EBSCO. We also used similar meanings for each keyword (eg, spoiled, corrupt, damaged, ruined, and expired for similar meanings of rotten) and linked them with “OR” and “AND” as query sentences (see Textbox 2). We exported each paper’s metadata, such as the title, year of publication, author, DOI, publisher, and keywords, and stored it to Google Spreadsheets. We used the PRISMA (Preferred Reporting Items for Systematic Reviews and Meta-Analyses) flow diagram to manage duplicate papers between databases and to screen review papers (see Figure 1) [31]. We excluded papers not meeting the eligibility criteria and found 19 eligible papers. After reviewing each paper to examine the research questions and objectives of prior studies, we found that no prior studies have reported how to support physically healthy older adults in determining the freshness of their diet using readily available equipment, such as a smartphone.

**Search query used in Scopus.**

TITLE-ABS-KEY(((“spoiled” OR “rotten” OR “corrupt” OR “damaged” OR “ruined” OR “expired”) AND (“food” OR “cooking” OR “kitchen” OR “food” OR “food” OR “meals” OR “kitchens” OR “kitchens” OR “food” OR “meals”) AND (“pick” OR “pick” OR “take” OR “take” OR “detects” OR “detects” OR “identifies” OR “identifies” OR “recognizes” OR “recognizes”) AND (“app” OR “application” OR “system” OR “platform” OR “apps” OR “applications” OR “systems” OR “platforms”))).

### App Development

**Figure 2:**
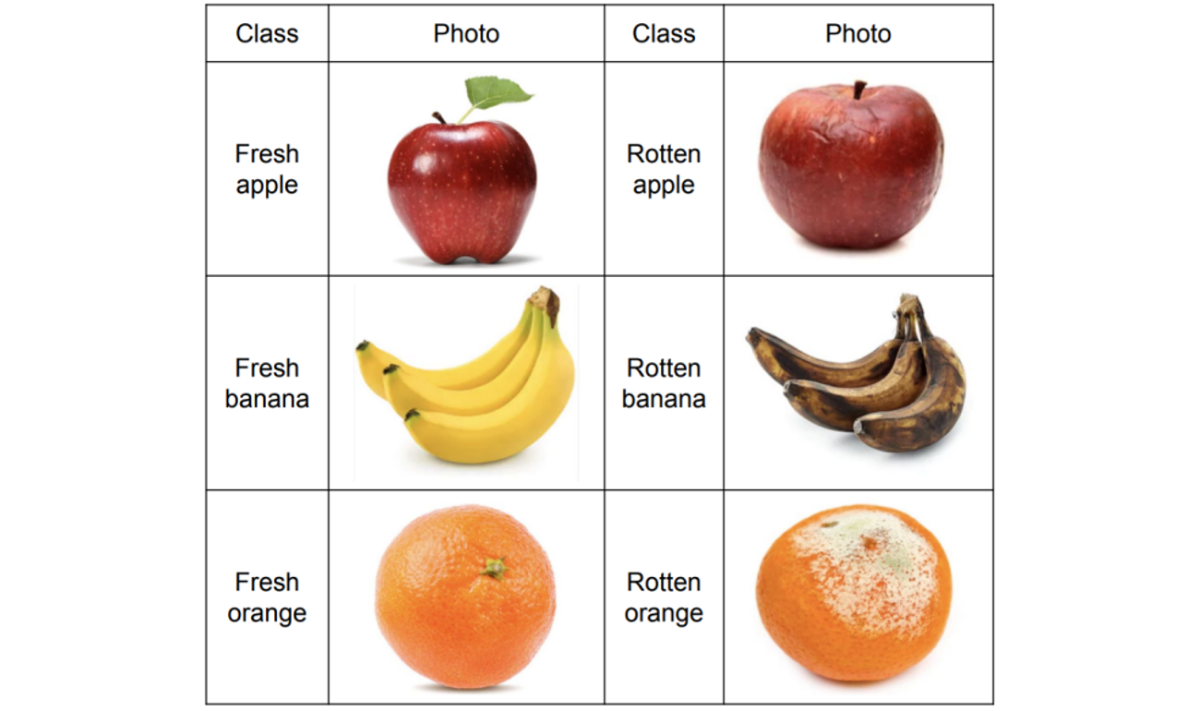
Pictures of apples, bananas, and oranges collected from Kaggle.

The objective of this study was to develop an app that determines whether an apple, a banana, or an orange is rotten just by taking a picture of the fruit using a smartphone camera. App development and evaluation procedures consisted of 4 steps: (1) collecting images, (2) training classification models, (3) developing an app, and (4) evaluating the models. First, we collected pictures required for training the food classification model. We collected pictures of fresh and rotten apples, bananas, and oranges from Kaggle, a platform that provides preprocessed data sets to data scientists: 2088 pictures of fresh apples, 1962 pictures of fresh bananas, 1854 pictures of fresh oranges, 2943 pictures of rotten apples, 2754 pictures of rotten bananas, and 1998 pictures of rotten oranges (see Figure 2). We uploaded and stored the collected pictures on the server for classification model training.

Second, we trained food picture classification models. To use only the features of the fruit in the picture for training, we segmented the pixels of the fruit from the background using Otsu’s [32] method. We stored the segmented pictures with the same size as the original pictures. When training the models, we used several pretrained residual networks (ResNets; eg, ResNet-50, ResNet-50V2, ResNet-101, ResNet-101V2, ResNet-152, ResNet-152V2, Inception-ResNet-V2) for feature extraction, reducing the time for model training and improving model performance [33]. Among the parameters used in the single fully connected layer, we set the activation function among sigmoid, rectified linear unit (ReLU), and tanh. We set the dropout rate to 0, 0.1, 0.2, 0.3, 0.4, or 0.5 and the number of neurons to 64, 128, 256, 512, or 1025. We randomly selected 100 combinations of the activation functions, the dropout rate, and the number of neurons. Among the combinations, the layer combination that showed the best performance consisted of a dense (neurons=128, activation=sigmoid) layer, a dropout (drop rate=0.2) layer, a dense (neurons=128, activation = sigmoid) layer, and a dropout (drop rate=0) layer.

**Figure 3:**
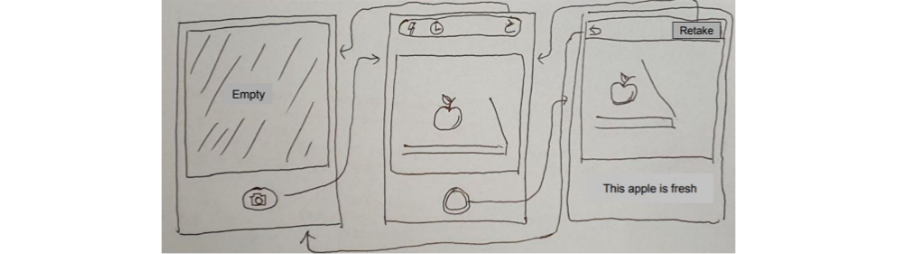
Low-fidelity prototype of the app, intentionally sketched by hand to emphasize the conceptual stage of design. Each box represents the shape of the smartphone running the app. Arrows depict screen transitions triggered by user interactions with specific elements on the screen. Low-fidelity prototypes are basic visual representations that do not incorporate high details or functionalities but are essential for rapid iterations and facilitate early discussions among researchers on design concepts, making hand-drawn sketches an ideal format for such type of prototype.

**Figure 4:**
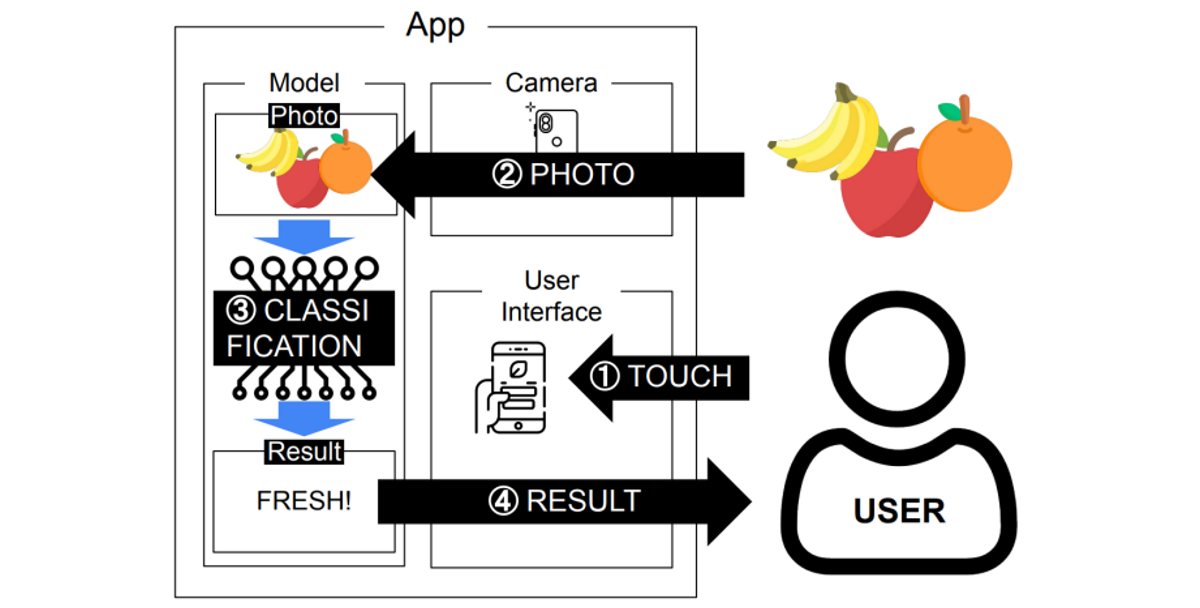
The 4 main features of the app. When a user takes a picture of a fruit, the picture is classified by the classification model and returned to the user.

**Figure 5:**
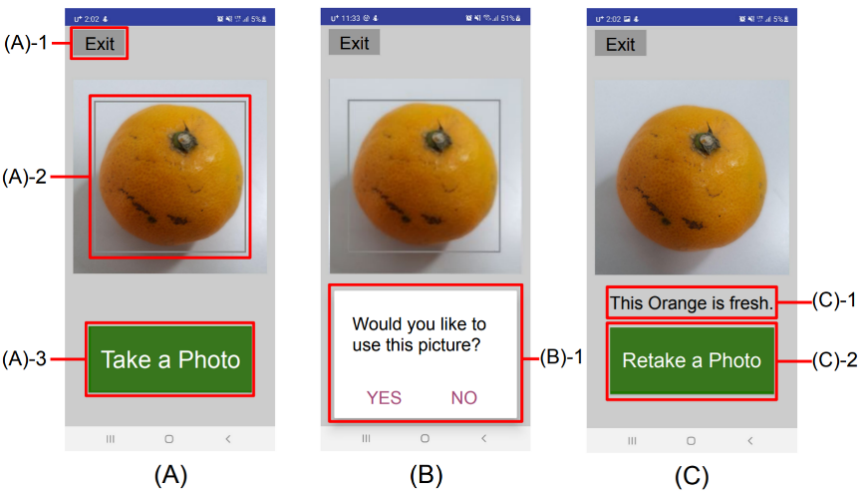
User interface screenshot. (A) Interface where the user takes a picture: (A)-1. Button to exit the app. (A)-2. Screen that shows what the camera is focusing on. (A)-3. Button to take a picture. (B) Interface for reviewing pictures taken by the user and deciding whether to use the pictures: (B)-1. Popup that asks the user whether to use a photo. (C) Interface for displaying the results of classifying the freshness of the fruit: (C)-1. Text that indicates the type and freshness of the photographed fruit. (C)-2. Button to return to the home screen of the app so that the user can take a picture of another fruit.

Third, we developed a smartphone app that uses a classification model that classifies fruits in pictures into fresh or rotten fruit. Android Studio [34] is the official integrated development environment for Android apps; the proportion of smartphone users in their sixties and older is steadily increasing, and the older they are, the more they use Android than iOS. Level 29 of the app is the minimum application programming interface (API) level that supports app download from Google Play, and the API level that can be operated on most smartphones was selected. We developed 3 functions: a function to take a picture using a smartphone camera, a function to input pictures into the classification function, and a function to show the classification result on the app screen. We created a low-fidelity prototype [35] to determine user tasks and 3 features of the app (see Figure 3). We added a camera function and set permissions (eg, camera, internal storage) for the function that would enable users to take pictures. The pictures taken are resized to 256 × 256 pixels to fit the input size of the trained classification model. The resized pictures are input to the converted classification model with the .h5 extension. Third, the app takes the output array from the classification model that consists of the probability value and displays one of the following texts: “The apple is rotten,“ ”The apple is fresh,“ ”The banana is rotten,“ ”The banana is fresh,“ ”The orange is rotten,“ and ”The orange is fresh“ (see Figures 4 and 5). To perform these functions, we added several buttons to allow users to interact with the app.

Lastly, we evaluated the performance of our trained models (eg, area under the curve [AUC], error rate). The model evaluation aimed to determine the best-performing model among the models developed using multiple backbone networks in the model training stage. To fix the metric to be used for model evaluation, we found the metric used to evaluate the classification model in each of the papers in the related area. A prior study used the *F*_1_-score, balanced accuracy, the error rate, and the AUC to evaluate the classification model [23]. After extracting the above metrics through 10-fold cross-validation, we found a significant difference between each model’s performance by using the Kruskal-Wallis test [36], a method that can be used when n≤30 for ≥3 classes. We performed this test to check whether using different backbone networks makes significant differences in model performance.

### Performance Evaluation

We evaluated the app’s performance to answer research questions 4 and 5. We performed a performance evaluation of the classification model used in the app, a quantitative evaluation using an after-scenario questionnaire [29], and a qualitative evaluation through semistructured interviews with the participants.

#### Recruitment

We recruited older adults, who are the target users of the app, as participants. We set eligibility criteria as follows: (1) 65 years of age or older, (2) those who have used a smartphone with a rear camera for at least 1 year, (3) those who have taken pictures with a smartphone at least once in the past 3 months, (4) those who could understand the contents of the questionnaire, (5) those who have lived in Korea for more than 5 years and can communicate in Korean, and (6) those who have not participated in this study before. However, we excluded participants unwilling to be research subjects or those with any chronic diseases [37]. We recruited participants using 3 methods: (1) recruiting through acquaintances, (2) recruiting through an institution used by older adults, and (3) recruiting through an online community used by older adults. When recruiting participants through acquaintances, we informed people [38] about the purpose of the study, the expected time required for the experiment, the eligibility of participants, and the reward. When recruiting through institutions, we collected the name, location, phone number, and email of the senior welfare center in Seoul and sent an email requesting to promote the experiment and share a promotional poster. When recruiting participants through an online community, we posted promotional posters by joining social network services, such as Open Kakao Talk, Naver Band, and Naver Cafe, which are thought to be used by older adults.

#### Study Procedure

We evaluated the usability of the app through an after-scenario questionnaire and semistructured interviews. We conducted an experiment by visiting each participant’s home. The researcher informed participants about the (1) purpose of the experiment, (2) purpose of using the app, (3) expected time required for the experiment, (4) function of the app, and (5) researcher (a brief introduction). After the participants agreed to get enrolled in the study, we instructed them about 3 tasks using the app: (1) run the app and review the screen, (2) a picture of a fruit using the app, and (3) review the text indicating whether the fruit in the picture taken is fresh. To conduct the experiment in the same environment as far as possible, we handed the smartphone with the app preinstalled to the participants. To minimize the impact of imaging angles, environments, illumination, cameras, and any other potential factors on the efficiency of classification, a standardized protocol was implemented across all experiments. For instance, each participant was asked to use the Samsung Galaxy Note 10 camera to photograph the fruits to ensure consistency in device specifications. The pictures were taken at a 45° angle under sufficiently bright light, such as in the kitchen or living room. Furthermore, by having participants align the fruits with the square gray lines shown in Figure 5, we ensured that the orientation and size of the fruits within the pictures did not affect the classification results. Each participant performed the given task with a fresh apple, a rotten apple, a fresh banana, a rotten banana, a fresh orange, and a rotten orange. When the participants completed their tasks, we asked them to fill out a questionnaire (see Textbox 3) and then interviewed them. The questionnaire consisted of questions regarding the experiences of the participants, considering the research questions, using a 5-point Likert scale ranging from 1 (strongly disagree) to 5 (strongly agree). When the interview was over, we stopped recording and paid Korean won 10,000 (approximately US $8) to each participant in cash. The questionnaire and the consent form were scanned and uploaded to Google Drive, along with the interview recording file. Finally, the interview recording file was transcribed, and we listened to the recording file again and corrected the script.

**After-scenario questionnaire for study participants using a 5-point Likert scale.**

Questions to answer the research questions (1 for strongly disagree to 5 for strongly agree):

1. I think it was easy to touch the app icon and review the first screen of the app.
2. I feel satisfied with the time it took to touch the app icon and review the app’s first screen.
3. I think it was easy to take a picture of the fruit.
4. I am satisfied with the time it took me to take a picture of the fruit.
5. I think it was easy to review the text indicating whether the fruit was fresh.
6. I feel satisfied with the time it took to review the text indicating whether the fruit was fresh.
7. I trust the text indicating whether the fruit is fresh.
8. Even if the app is paid, I plan to use it.

### Data Analysis

**Figure 6:**
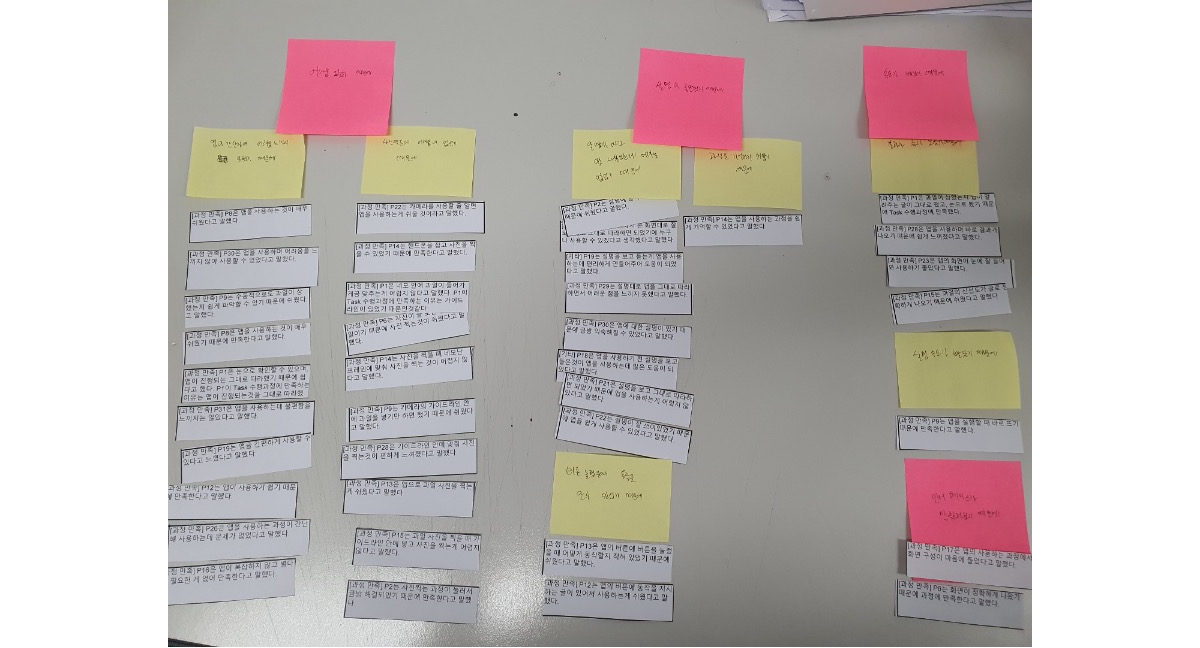
Qualitative data analysis process, a vital step in numerous research methodologies. White notes represent codes written by the researcher for excerpts from interview transcripts. These codes highlight key ideas or concepts within the data. Yellow notes signify emergent themes identified through an initial coding stage. Here, similar or related codes are grouped together to synthesize broader patterns within the data. Red notes denote overarching themes or categories derived from a subsequent grouping of the yellow notes. This final stage reflects a higher level of data abstractions, moving from specific codes to more general thematic constructs.

We quantitatively and qualitatively analyzed the participants’ responses. We removed every participant’s identification information and assigned them a new one (eg, P1, P2). Next, we calculated the questionnaire responses’ mean (SD) scores for quantitative analysis. For qualitative analysis, we used open-coding methods that highlighted meaningful remarks within scripts. We highlighted statements that included important information (eg, participants’ experiences, preferences) in each script [39]. For each highlighted remark, we assigned a label to summarize the remark. We printed all the labels and inductively grouped them to extract important themes that answered our research questions (see Figure 6) [40]. For qualitative research, affinity diagramming, a technique using sticky notes, is a prevalent practice due to its inherent flexibility and tangibility, which facilitates the organization of thoughts and findings in a spatially representative way. The ability to physically manipulate and rearrange data points is a key advantage of affinity diagramming, fostering deeper thematic analysis and the identification of emergent relationships and patterns. This technique also promotes collaborative discussions among researchers by providing a clear visual representation of the data analysis process.

### Ethical Considerations

All experimental procedures were approved by the Institutional Review Board (IRB) of the University of Seoul (IRB ID: 2021-06-001-001). Prior to the experiment, each participant provided informed consent by signing a consent form about participating in the study and recording the interview, which also included a clause allowing the use of collected data for secondary analyses without additional consent. To protect the privacy of collected data, all participant data were anonymized and stored on a secure drive, which only authorized personnel have access to. After completing the interviews, all participants received a cash compensation of South Korean won 10,000 (approximately US $8) for their participation.

## Results

### Participant Demographics

Table 1 shows the details of the 26 participants enrolled in this study.

**Table 1:** Participant (N=26) demographics.

Participant ID	Age (years)	Gender	Take pictures of food often (1: strongly disagree to 5: strongly agree)	Usually check that food is fresh before eating it (1: strongly disagree to 5: strongly agree)
P1	65	Female	Not reported	Not reported
P2	70	Male	Not reported	Not reported
P3	66	Female	Not reported	Not reported
P4	66	Female	Not reported	Not reported
P5	65	Male	Not reported	Not reported
P6	69	Male	1	4
P7	73	Female	2	5
P8	73	Female	2	5
P9	65	Female	2	5
P10	65	Male	1	5
P11	74	Male	3	5
P12	69	Female	1	3
P13	69	Male	2	4
P14	65	Male	1	5
P15	69	Male	2	4
P16	76	Male	5	5
P17	69	Male	1	5
P18	76	Male	1	1
P19	67	Female	1	5
P20	72	Male	1	4
P21	68	Female	3	5
P22	70	Male	3	4
P23	65	Male	1	4
P24	66	Female	3	4
P25	68	Male	3	4
P26	66	Female	3	5

### Model Performance, Survey Results, and Interview Results

The results of our study were classified into (1) performance evaluation results of the trained model during app development, (2) quantitative analysis results of responses collected through the survey, and (3) qualitative analysis results of responses collected through interviews.

#### Model Performance

We trained each classification model using 7 ResNets as a backbone and compared their performance using 10-fold cross-validation to review whether there was a significant difference in performance. ResNet-101 showed the highest performance, on average (see Table 2). In this study, the optimal architecture for the fully connected layers was identified as a sequence containing a dense layer with 128 neurons and sigmoid activation, a dropout layer with a drop rate of 0.2, and another dense layer with 128 neurons also using sigmoid activation. The final layer in the sequence was a dense layer without dropout. The learning rate was set to 0.001. The training process was configured with 1000 epochs, incorporating an early stopping mechanism with a patience parameter of 20. Whether the model trained using ResNet-101 and the rest of the backbone network showed a significant performance difference was verified using the Kruskal-Wallis test [36]. As a result, ResNet-101 showed a significant performance difference from ResNet-50, ResNet-50V2, ResNet-101V2, and Inception-ResNet-V2, but there was no significant difference between ResNet-152 and ResNet-152V2. Therefore, although the trained model using ResNet-101 showed the highest performance among the 7 backbone networks, there was no significant performance difference when compared with ResNet-152 and ResNet-152V2.

**Table 2:** AUC^a^, error rate, balanced accuracy, and *F*_1_-score of ResNet^b^-101 after training the model using each backbone network with 10-fold cross-validation.

Backbone	AUC	Error rate	Balanced accuracy	*F*_1_-score
ResNet-50	0.9533	0.3589	0.7523	0.7615
ResNet-50V2	0.9456	0.3591	0.7409	0.7495
ResNet-101	0.9663^c^	0.3216^c^	0.7949^c^	0.8003^c^
ResNet-101V2	0.9467	0.3533	0.7403	0.7494
ResNet-152	0.9607	0.3475	0.7783	0.7833
ResNet-152V2	0.9606	0.3250	0.7806	0.7884
Inception-ResNet-V2	0.9210	0.4097	0.6821	0.6932

^a^AUC: area under the curve.

^b^ResNet: residual network.

^c^The highest performance shown by ResNet-101.

#### Survey Results

We obtained the mean (SD) of the demographic information and the participants’ responses to each survey question. A total of 32 responses were obtained; however, 6 (23.1%) participants (P3-P5, P7, P10, and P11) did not meet the criteria for participation, so their responses were excluded. According to the demographic information shown in Table 1, the mean age of the 26 (100%) participants was 68.69 (SD 3.47) years, of which 15 (57.7%) participants were male and 11 (42.3%) were female. The participants generally had a positive perception of the app (see Table 3). They generally agreed that it was easy to run the app and review its first screen, and they were satisfied with the time it took to run the app and review the first screen. In addition, they thought that taking pictures of fruits is easy, and they were satisfied with the time required to take a picture. They also thought that reviewing the text indicating whether the fruit is fresh is easy, they were satisfied with the time duration from taking a picture to reviewing the text, and they trusted the result displayed on the app. However, they did not intend to use the app if it is a paid app. According to the results, the freshness of fruits can be efficiently reviewed with the app.

**Table 3:** Survey results showing the perceptions of older adults about the app.

Question number	Survey question (1: strongly disagree to 5: strongly agree)	Mean (SD)
1	Do you agree that running the app and reviewing its first screen is easy?	4.58 (0.70)
2	Do you agree that you were satisfied with the time it takes to run the app and review its first screen?	4.77 (0.51)
3	Do you agree that taking pictures of fruits is easy?	4.81 (0.49)
4	Do you agree that you were satisfied with the time required to take a picture of a fruit?	4.81 (0.40)
5	Do you agree that reviewing the text indicating whether the fruit is fresh is easy?	4.73 (0.67)
6	Do you agree that you were satisfied with the time duration from taking a picture of a fruit to reviewing the text indicating whether the fruit is fresh?	4.88 (0.33)
7	Do you agree that you trust the result displayed on the app?	4.38 (0.90)
8	Do you agree that you intend to use the app even if you have to pay for it?	2.73 (1.28)

#### Interview Results

We analyzed the interview results qualitatively to answer research questions 4 and 5:

- Research question 4.1: Why are older adults satisfied with the process of using the app?
- Research question 4.2: Why are older adults dissatisfied with the process of using the app?
- Research question 4.3: Why are older adults satisfied with the time it takes to complete the task?
- Research question 4.4: Why are older adults dissatisfied with the time taken to complete the task?
- Research question 4.5: What makes older adults satisfied using the app?
- Research question 4.6: What makes older adults dissatisfied with using the app?
- Research question 5.1: Who are the potential users of the app?
- Research question 5.2: Why did older adults suggest them as potential users?
- Research question 5.3: Why can older adults trust the app?
- Research question 5.4: Why can older adults not trust the app?
- Research question 5.5: What features would older adults like to add to the app?
- Research question 5.6: Why do older adults use the app even if it is a paid app?

Since no older adults responded that they were dissatisfied with the time taken to use the app, we excluded the answer to research question 4.4 from the results.

#### Reasons Why Older Adults Are Satisfied or Dissatisfied With the Process of Using the App

Participants stated that there are several reasons why they were satisfied with the process of using the app. For example, 10 (38.5%) participants reported that the app is simple and easy to use:

I do not have to do something in several steps, I just take pictures.

P10

Similarly, 7 (26.9%) participants responded that they had no difficulty taking pictures:

It was not difficult for me to take pictures using the app.

P22

Six participants said that it is not difficult to use the app as the description is sufficient:

The description of the app was very helpful. Without this explanation, I would have taken the wrong picture, and I do not think I would get the desired result.

P12

Four participants responded that the app works fast, so they immediately saw a text indicating the freshness of the fruit on the app:

As soon as I took a picture, the text was immediately visible, so I could check whether it was fresh or not, which was nice.

P22

Two participants said they were satisfied with the interface:

I think I can give it almost 90 out of 100 points for touching and reviewing the screen of the app. The screen composition is almost 90 points.

P11

However, some participants still found it difficult to take pictures or check fruit freshness. For example, 2 (7.7%) participants reported that taking pictures using the app is difficult:

It would be much better if I could just take pictures, whether from close or far away. But when I tried to fit the object inside the square, my hand was shaking, which made it difficult to take the photo.

P12

Two other participants reported that they could not quickly check the text indicating freshness:

The speed at which I can check whether the fruit is fresh or not will still be around 3 out of 10. I could not check it right away.

P3

#### Reasons Why Older Adults Are Satisfied With the Time It Takes to Use the App

Participants were generally satisfied with the time it took to use the app. For example, 13 (50%) participants said that it does not take long to use the app:

When I touched the app, it did not feel like it took that long, such as buffering, and the screen popped up immediately.

P23

Two participants responded that they felt it is easy to use the app:

I felt satisfied because I understood it quickly.

P7

One participant said that she was satisfied with using the app because it actually takes a long time:

I liked being able to use the app at my own pace rather than using it quickly.

P3

#### Reasons Why Older Adults Are Satisfied or Dissatisfied With Using the App

Participants responded that they were satisfied with the app because it is visually pleasing and simple. For example, 16 (61.5%) participants reported that the interface of the app is visually satisfactory:

It was easier because the font size was okay and the freshness was simply indicated.

P14

In addition, 8 (30.8%) participants reported that they had no difficulty taking pictures using the app:

The app is so easy to use because I can take a picture right away. When taking pictures of distant objects with a general camera, I need to adjust the distance and adjust the direction, but with this app, it was easy.

P15

Six participants responded that the process is simple and easy to use:

This app was uncomplicated and easy to use.

P15

In addition, 2 (7.7%) participants said that the app is easy to use because it does not require any authentication, 2 (7.7%) participants said they liked being able to check freshness using the app, 2 (7.7%) said that the app could provide health-related information, and 2 (7.7%) responded that they enjoyed using the app:

For example, it does not require authentication or anything like that, just taking a picture.

P10

I think that using the app is easier than tasting or smelling or anything like that. In order to check the smell or taste, I have to come in direct contact with the food now, so there is such an inconvenience.

P14

The most important thing is to check things related to my health.

P2

With my children, I check whether the fruit is rotten or not, and there are things like that. That could be fun.

P15

However, some participants reported that they had difficulty using the camera or that the app is too simplistic. For example, 7 (26.9%) participants responded that they felt troublesome while using the app:

When we look at the fruit with our own eyes, we clearly identify whether it is fresh. Other methods make me inconvenience.

P9

Four participants responded that the interface is still small:

It would be better if the camera screen were bigger.

P17

Furthermore, 2 (7.7%) participants said that using the camera is uncomfortable, another 2 (7.7%) said the app’s design is too simple, and 2 (7.7%) said the app’s functionality is too simple:

The app was similar to the camera, but the camera app is better because it is good to focus on an object.

P13

I think the app needs to be a little more sophisticated. The elements are so angular and seem like a little basic app.

P16

The app is too simple. I have no intention of using the app because of the few features.

P5

One participant said that using the app might appear to question the freshness of the food items:

I think it would be a bit strange to take a picture while having a meal together. But if the app has become a bit more popular, I think it is okay to try it once.

P22

#### What Older Adults Want From Using the App

Participants wanted the app to inform them of fruit freshness in various ways, and they also wanted the app’s detection accuracy to be improved. For example, 10 (38.5%) participants said they wanted to learn more about using the app:

It would be nice to check the freshness of not only fruits but also the kinds of herbs and various food items.

P15

Seven participants said they wanted the app to be easier to use:

If the fruit pictured here is rotten, I think the result should be displayed in red, or if it is fresh, it should be displayed in blue. It is fine now, but would it not be better if the app guided me with red, blue, and yellow colors?

P25

Three participants said they wanted the app to give accurate freshness:

I think the results are inaccurate. I will use it if the accuracy is higher.

P9

Two participants said they wanted to check freshness in a variety of ways:

The app only checks the freshness by the shape of the fruit. Can we add a function using a smell or something like a taste?

P20

One participant said that they would like to see an additional way to earn points as they use the app:

It would be nice if there were merits, such as earning points the more I use the app.

P2

In addition, participants suggested some features for the app. They wanted it to be easier to take pictures or to obtain more information about the fruit. For example, 3 (11.5%) participants said that they would like the app to automatically focus on the fruit when they take a picture of a fruit:

Rather than focusing the camera on an object, it will be convenient to know the result just by being on the screen, no matter how the camera captures the fruit.

P5

Two participants said they would like to add a feature that provides more information about the fruits they have photographed:

So, if I just take a picture of the fruit, the app has to tell me all the information about the fruit.

P5

#### Potential Users of the App

Participants said that they would be able to recommend the app to their acquaintances, homemakers, and even young people. For example, 4 (15.4%) participants said that their acquaintances could use the app, while 1 (3.8%) said that they could recommend the app to homemakers:

I will try to share it with the church’s female teachers and priests of my age.

P17

I think homemakers will use it a lot.

P23

Four participants said that they could recommend the app to people over the age of 50 years, including older adults:

I think people who are as old as us or older can try it.

P12

In contrast, 2 (7.7%) participants said that they could recommend the app to younger people:

In my opinion, young people in their twenties and thirties have no experience checking the freshness, so that the app may be widespread.

P21

Three participants said that using an app to check freshness at a fruit shop would give customers peace of mind:

Individuals, of course, need to check the freshness at home, but a fruit store asks customers to check the freshness of the fruit using an app, and if the fruit is fresh, then customers can buy with confidence.

P20

One participant said that she could recommend the app to health-conscious people:

I can recommend an app to anyone sensitive or unusually picky about health and food items.

P4

In contrast, 1 (3.8%) participant said that they did not intend to recommend the app to others:

I do not intend even to recommend the app to others.

P10

Participants said that potential users of the app would quickly learn how to use it. For example, 7 (26.9%) participants said the app is simple, which would make it easier for potential users to use it:

Rather than saying that this is easy to learn, is it not that everyone can do it right away? Because the app is very simple and easy. I think this can be done whenever a person feels it is necessary without the need to learn anything.

P20

Three participants said that potential users would be able to use the app to purchase fresh food items:

Since homemakers buy the most ingredients at the mart, I can recommend it to housewives.

P23

Two participants said that potential users would be able to use the app to tell whether a food item is visually fresh:

When they say they are unsure if the fruit is fresh by looking at it, would it not be possible to check it through the app?

P19

One participant said that if there is a good app, it should be shared with potential users, while another said that a potential participant would be interested in trying the app out of curiosity:

The good thing is that we all have to share.

P16

I will probably try it at a mart or a fruit store mainly out of curiosity.

P21

#### Why Older Adults Trust or Distrust the App

Participants were less skeptical about the app and tended to believe it, and only a small fraction of participants said they would compare it with their own thoughts. For example, 9 (34.6%) participants said that they could trust the app because it is just an app to tell them about freshness:

I did not think about why, and if I do not trust my cell phone, what do I believe? I just believed it.

P7

In addition, 2 (7.7%) participants said that they could trust the app if they used it frequently, while another 2 (7.7%) said that the results were the same as they thought:

If I use the app once or twice, and the results are correct, then I should trust the app.

P15

When I look at the fruit, if it looks like the fruit is rotten and matches the result, then I can trust the app.

P16

However, 7 (26.9%) participants said they trusted their senses more than the app:

Would it not be more accurate to check with my own eyes rather than the app?

P19

Three participants responded that their thoughts on the freshness of the fruits and the results were different:

In my opinion, the fruit is not fresh, but the app says the fruit is fresh.

P9

Six participants said that they trusted their senses more because they had not used the app much:

If the results are certain when I use the app, I will believe it from now on, but this is the first time I have used it.

P15

#### Why Older Adults Would Use or Not Use the App If They Need to Pay

Most of the participants said they would not use paid apps and were reluctant to pay. For example, 8 (30.1%) participants said that they did not feel the need to use a paid app:

I do not think I need to use an app that requires money.

P20

Four participants responded that they found it difficult to use the app:

It is inconvenient to use such an app; I do not know whether it is paid or free, and I do not need it, too.

P18

Furthermore, 3 (11.5%) participants said they prefer free apps, while 2 (7.7%) said that using paid apps requires money:

So, first of all, it is better to be free.

P10

Older adults prefer to save money rather than not believe the app.

P7

In contrast, 8 (30.1%) participants responded that if they needed the app, they would pay to use it, depending on the situation:

If I thought I really needed the app, I would use it often.

P22

## Discussion

### Principal Findings

The results of this study showed the behaviors and perceptions of older adult participants when using our app. The survey responses revealed their positive perceptions about the app’s ease of use and their satisfaction with the time taken to complete tasks. However, the survey also showed a low willingness to pay for the service among older adults. Interviews provided further insights, revealing participants’ appreciation for the app’s usability, rapid result generation, and straightforward interface. Conversely, some participants expressed a desire for more features, while others raised concerns regarding trust in the app. In the following sections, we discuss findings based on the results, the limitations of our study, and future work.

#### Factors Affecting Satisfaction When Using the App

Some participants mentioned that they were satisfied, even though it took a long time to review the freshness of rotten fruits using a smartphone app. In general, it is thought that the shorter the time it takes for users, the more satisfied they will be; however, 1 participant said that the longer it takes, the more they feel satisfaction. That is, when older adults use mobile health apps for health management purposes, the factors that have the greatest influence on them differ with time. According to a study that found the dissatisfaction of older adults who watch YouTube using smartphones, it is the data plan that older adults feel most dissatisfied with [41]. This finding is similar to our results. When older adults use mobile health apps, the most unsatisfactory thing for them may be that they need to pay costs or equivalents. We found that some older adults enjoy the process of using apps rather than reaching their own goal. According to a study, the reasons why older people use YouTube are to gain knowledge about political and social issues, travel information, food recipes, and health information [41]. In other words, it can be seen that when older adults use a specific app, they can also enjoy the process of achieving that purpose. Harris et al [42] suggested that perceived usefulness, ease of use, and enjoyment are significant motivators for older adults to adopt new technologies. Consequently, the enjoyment derived from using smartphone apps may potentially mitigate the initial hurdles faced by this group in technology adoption. Additionally, clear instruction materials and reduced costs have been identified as further facilitators for older adult engagement with deep learning and smartphone-based technologies [42].

#### Potential Users of the App

Some participants responded that an app for classifying fruit freshness would be necessary for young people who lack experience in distinguishing rotten fruits by sense, instead of older adults. These young people may consume rotten food items due to their lack of experience in checking freshness, even if their cognitive function is normal. According to a study on the kinds of bacteria that cause food poisoning and are found on the hands of elementary school students, coliforms were detected the most [43]. Based on this, it seems that the age groups most likely to experience health problems due to food poisoning are students rather than older adults, so they may be potential users of the app.

#### Factors Affecting Participants’ Confidence in the App

Some participants responded that they trusted the app results because it was an app running on a smartphone, contrary to our belief that older adult users would need something to convince them to trust the app. We discovered that some people might not need a reason to believe and use something. A study examining the effect of trust in technology on the adoption of technology before revealing why older adults trust and use an app without any rationale showed that an app’s background factors greatly influence the adoption of technology [44]. Likewise, we assumed that some older adults who used the app without evidence would not just trust the app but also another technology that they think will work behind the smartphone’s system.

### Limitations and Future Work

We developed an app that detects rotten fruit for the health management of older adults and investigated their experiences from various aspects. However, future work is still needed to gain deeper insight into the topics discussed. First, we developed a smartphone app that determines whether a fruit is fresh when older adults take a picture of it, but the app does not include a function to distinguish the freshness of food items other than the 3 fruits (apples, bananas, and oranges). According to the results, 8 participants wanted to know the freshness of food items other than the 3 fruits. It might be important to determine the freshness of a wider variety of food items, such as vegetables, herbs, and meat, to contribute to preventing older adults from experiencing health problems due to eating rotten food items. However, to determine the freshness of food items other than the 3 fruits, a large number of images are required, which requires a lot of time and resources. According to a previous study that proposed a visual-based method for detecting rotten food items, rotten food items have common visual characteristics [45], using which might enable the determination of the freshness of more diverse food items without collecting corresponding food image data.

Second, we used 7 ResNets as backbone networks to create a model that can distinguish the freshness of 3 fruits. We selected ResNet-101 with the highest classification performance among the 7 networks. However, we did not use several other backbone networks. Using more diverse backbone networks, including the recently proposed backbone network, might improve the performance of the model. Recently proposed models in a classification problem using ImageNet showed more than 10% higher accuracy than the backbone network used in our study [46,47]. The performance of classifying rotten food images might be greatly improved using the recently proposed network.

Third, we analyzed the collected interview scripts using open coding, but 2 or more researchers did not participate in this step. Participation of more than 2 open coders is required to enrich the quality of the results and reduce the bias that may occur when a single coder is performing the analysis.

Regarding future work, first, to help older adults use the app for determining the freshness of various food items and not eat rotten food, researchers need to add a function that distinguishes the freshness of food items other than the 3 fruits. Second, to enable older adults using the app to more accurately check the freshness of food items, researchers may adopt recently developed networks that show high performance in image classification as backbone networks. Researchers may use a pretrained image processing transformer [48] by fine-tuning it with the image data we collected. We may also extend the possible food categories by using generalized zero-shot learning [49]. Third, for app improvement, data collected from a questionnaire and interviews should be analyzed by 2 or more coders to guarantee the reliability of the findings of this study.

### Conclusion

The goal of this study was to help older adults avoid eating any rotten food items and not suffer from health problems. We established research questions and goals based on the limitations of prior studies where researchers created and evaluated tools for detecting rotten food items. To achieve the goals, we found the highest-performing classification model among various backbone networks by training a model to determine whether the targeted fruit was rotten. The trained model was used for developing a smartphone app. The findings of this study with older adult participants revealed that the usability of the app and the older adults’ perceptions about the app are positive, but they tend to feel reluctant to use the app if they need to pay for it. We also found how to design and evaluate an app using AI models targeting older adults by uncovering their perceptions about the app. Our study contributes to the research community by revealing which of the 7 pretrained backbone networks shows the highest performance on the ImageNet classification problem for determining whether the targeted fruit is rotten. We hope our proposed app enables older adults to identify rotten food items efficiently for maintaining their health.

## Abbreviations

AI: artificial intelligence
API: application programming interface
AUC: area under the curve
CNN: convolutional neural network
FTIR: Fourier transform infrared spectroscopy
PRISMA: Preferred Reporting Items for Systematic Reviews and Meta-Analyses
ResNet: residual network
SVM: support vector machine

## Data Availability

The data sets generated and analyzed during this study are available from the corresponding author upon reasonable request.
